# Novel Semi-Synthetic Cu (II)–Cardamonin Complex Exerts Potent Anticancer Activity against Triple-Negative Breast and Pancreatic Cancer Cells via Inhibition of the Akt Signaling Pathway

**DOI:** 10.3390/molecules26082166

**Published:** 2021-04-09

**Authors:** Md Shahadat Hossan, Mohammed Khaled Bin Break, Tracey D. Bradshaw, Hilary M. Collins, Christophe Wiart, Teng-Jin Khoo, Ahmed Alafnan

**Affiliations:** 1 School of Pharmacy, University of Nottingham, University Park, Nottingham NG7 2RD, UK; hilary.collins@nottingham.ac.uk; 2 Department of Pharmaceutical Chemistry, College of Pharmacy, University of Hail, Hail 81411, Saudi Arabia; 3 Centre for Natural and Medicinal Product Research, School of Pharmacy, University of Nottingham Malaysia, Semenyih 43500, Malaysia; Christophe.Wiart@nottingham.edu.my (C.W.); tengjin.khoo@nottingham.edu.my (T.-J.K.); 4 Department of Pharmacology and Toxicology, College of Pharmacy, University of Hail, Hail 81411, Saudi Arabia; a.alafnan@uoh.edu.sa

**Keywords:** cardamonin, complex, cytotoxicity, Akt

## Abstract

Cardamonin is a polyphenolic natural product that has been shown to possess cytotoxic activity against a variety of cancer cell lines. We previously reported the semi-synthesis of a novel Cu (II)–cardamonin complex (**19**) that demonstrated potent antitumour activity. In this study, we further investigated the bioactivity of **19** against MDA-MB-468 and PANC-1 cancer cells in an attempt to discover an effective treatment for triple-negative breast cancer (TNBC) and pancreatic cancer, respectively. Results revealed that **19** abolished the formation of MDA-MB-468 and PANC-1 colonies, exerted growth-inhibitory activity, and inhibited cancer cell migration. Further mechanistic studies showed that **19** induced DNA damage resulting in gap 2 (G2)/mitosis (M) phase arrest and microtubule network disruption. Moreover, **19** generated reactive oxygen species (ROS) that may contribute to induction of apoptosis, corroborated by activation of caspase-3/7, PARP cleavage, and downregulation of Mcl-1. Complex **19** also decreased the expression levels of p-Akt and p-4EBP1, which indicates that the compound exerts its activity, at least in part, via inhibition of Akt signalling. Furthermore, **19** decreased the expression of c-Myc in PANC-1 cells only, which suggests that it may exert its bioactivity via multiple mechanisms of action. These results demonstrate the potential of **19** as a therapeutic agent for TNBC and pancreatic cancer.

## 1. Introduction

Cancer is a major health problem worldwide with ~17 million new cases and 9.6 million deaths in 2018. It is predicted that the number of new cases will increase to 27.5 million cases by the year 2040. Breast cancer is one of the most common types of cancer globally, with ~2 million cases reported in 2018. A subgroup of breast cancer known as triple-negative breast cancer (TNBC) represents 15–20% of all breast cancers and is characterised by the lack of oestrogen receptor (ER) and progesterone receptor (PR) expression, in addition to the lack of human epidermal growth factor receptor (HER2) amplification. TNBC is of particular concern as it displays poor prognosis due to its inherently aggressive nature and lack of recognised molecular targets for therapy. Pancreatic cancer is another cancer phenotype that is highly lethal and is a major cause of cancer-related mortality with an overall 5 year survival of <7%. The appalling prognosis that is associated with pancreatic cancer is partially due to its aggressive nature and difficult early diagnosis. There is currently a lack of efficient treatments for TNBC and pancreatic cancer; therefore, further research is required in order to develop more effective treatments for these types of cancer [1,2,3,4,5].

Natural products have historically provided an important source of bioactive compounds and have been heavily studied for the purpose of developing effective anticancer agents. Polyphenols are a family of natural products that are widely abundant in plants and display anticarcinogenic properties, resulting in a large number of studies being dedicated to developing polyphenols into effective anticancer agents. Cardamonin is a polyphenolic natural product that belongs to a larger family of compounds known as chalcones and was found to exhibit pronounced cytotoxic activity against a variety of cancer cell lines such as A549, M14, U266, MDA-MB-231, HK1, and MCF-7 [6,7]. Moreover, there were many studies that attempted to investigate cardamonin’s mechanism of action where it was found to exert its cytotoxic activity via targeting a variety of signalling pathways such as JNK/FOXO3a, Wnt/*β*-catenin, and mTOR cascades [8,9,10].

**Figure 1:**
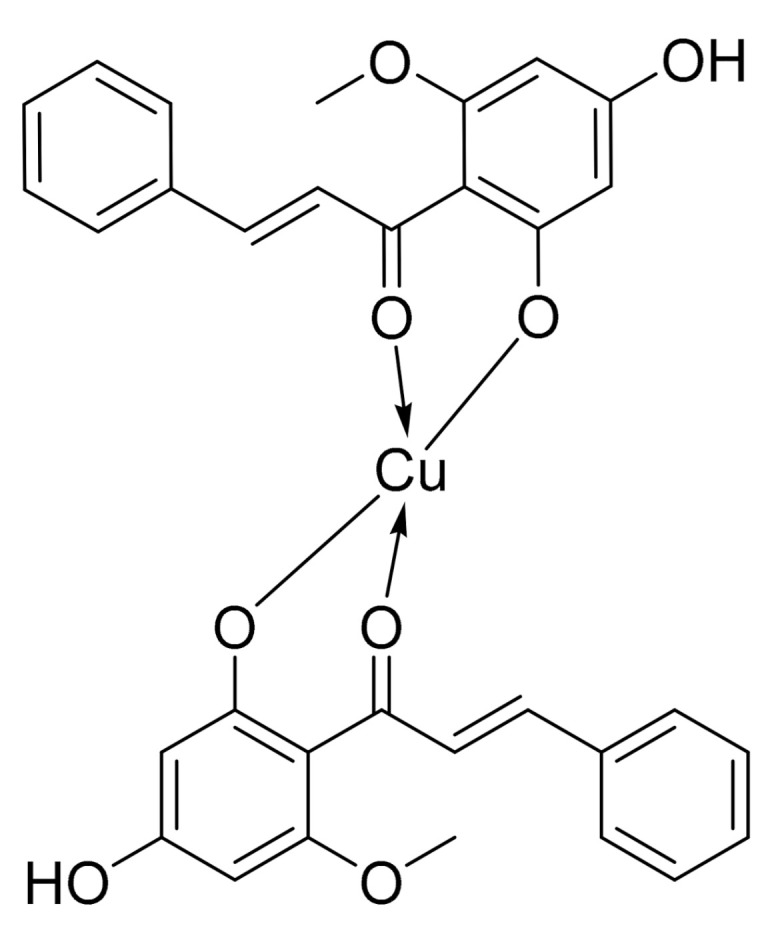
Structure of Cu (II)–cardamonin complex (**19**)**.**

As part of our efforts to discover and develop novel anticancer agents, we previously synthesised several analogues of cardamonin and discovered a highly active analogue [11]. This active analogue was the Cu (II)–cardamonin complex (**19**) (Figure 1) which displayed cytotoxic activity against lung cancer and nasopharyngeal carcinoma cell lines. However, to confirm the potential of **19** as a promising anticancer agent, we still have to investigate its cytotoxic activity against other types of cancer and elucidate in detail its mode(s) of action.

Therefore, the main aim of the present study was to examine the cytotoxic activity of **19** against intractable MDA-MB-468 (TNBC) and PANC-1 (pancreatic) cancer cell lines to further explore the potential of **19** as an effective anticancer agent and investigate its mode(s) of action. The study employed a wide range of biological assays to fully assess the antitumour activity of **19,** ranging from cell viability, apoptosis, and migration assays to cell-cycle and Western blot analysis. Herein, we describe the potential of the novel semi-synthetic compound, **19**, as a promising anticancer agent against TNBC and pancreatic cancer, in addition to reporting a detailed description of its mechanisms of action.

## 2. Results and Discussion

### 2.1. Compound ***19*** Inhibited the Growth and Migration of MDA-MB-468 and PANC-1 Cancer Cells

**Figure 2:**
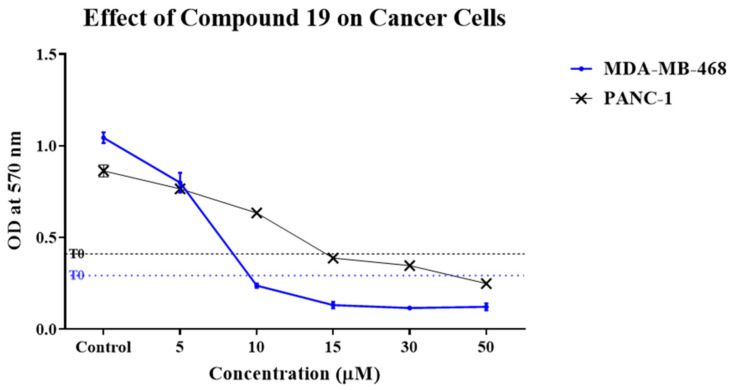
Representative dose–response profiles demonstrating growth-inhibitory effects of **19** against MDA-MB-468 and PANC-1 cells. The assay was repeated at least three times. Points and error bars refer to the mean ± SD.

We were previously able to synthesise a highly active Cu (II) complex of cardamonin (**19**) which demonstrated potent cytotoxic activity against A549 and HK1 cancer cell lines [11]. In order to further assess the cytotoxic activity of **19**, we examined its effect against MDA-MB-468 (TNBC) and PANC-1 (pancreatic) cancer cell lines. The effect of **19** on the cancer cell lines was initially investigated via 3-(4,5-dimethylthiazol-2-yl)-2,5-diphenyltetrazolium bromide (MTT) cell viability assay, and the results are summarised in Table 1. Compound **19** demonstrated potent growth inhibitory activity against both MDA-MB-468 and PANC-1 cells with 50% Growth inhibition (GI_50_) values of 6.14 µM and 12.48 µM, respectively, and it decreased their viability in a dose-dependent manner (Figure 2). Moreover, **19** showed an almost sixfold enhancement in activity against MDA-MB-468 cells relative to its parent compound, cardamonin, while a slight enhancement was observed against PANC-1 cells. This indicates that complexation of cardamonin to Cu^2+^ was a successful technique to enhance the natural product’s bioactivity. The enhanced bioactivity of **19** relative to cardamonin may be explained by Overtone’s concept and chelation theory which states that lipid cell membranes generally favour the passage of lipophilic compounds, and chelation/complexation enhances lipophilicity by allowing the delocalisation of π electrons over the whole chelate ring, while polarity of the metal ion is reduced due to its positive charge being partially shared with the donor groups [12]. Therefore, **19** demonstrated higher bioactivity than cardamonin probably due to its enhanced penetration ability into the cancer cells. Compound **19** was also screened against human normal lung fibroblasts (MRC-5) in order to investigate the compound’s selectivity toward cancer cells. Results of the screening showed that **19** exhibited 5.3- and 2.6-fold selectivity toward MDA-MB-468 and PANC-1 cancer cells relative to non-transformed MRC-5 cells, respectively. Moreover, **19** showed more cancer selectivity than cardamonin, especially in the case of MDA-MB-468 cells. Overall, results showed that **19** was more active toward MDA-MB-468 cells than PANC-1 cells, exhibited superior cytotoxic activity relative to its parent compound, cardamonin, and demonstrated cancer selectivity.

**Figure 3:**
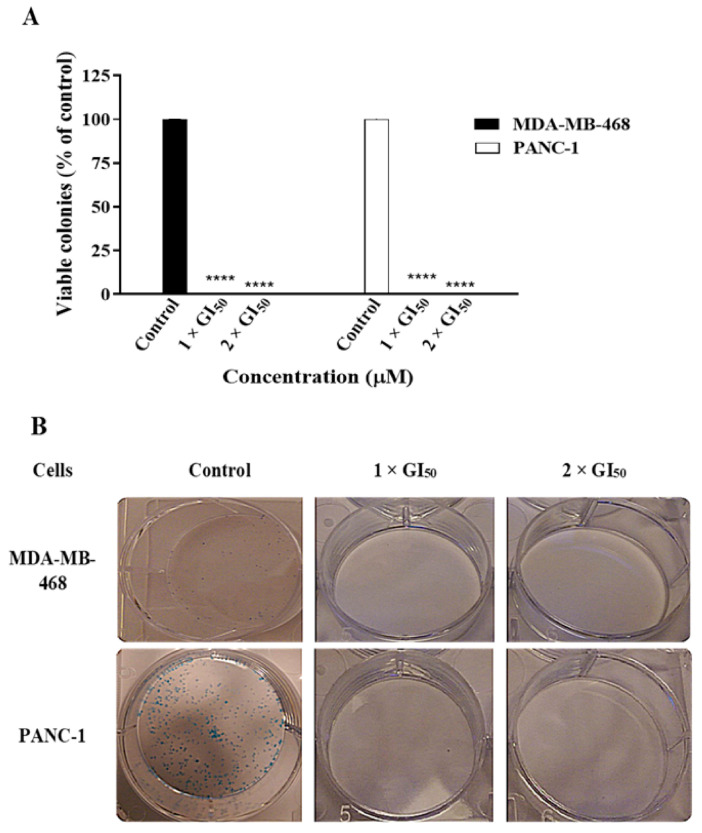
(**A**) Compound **19** completely inhibited the formation of colonies by MDA-MB-468 and PANC-1 cells after 24 h of treatment at concentrations of 1 × GI_50_ and 2 × GI_50_. The assay was repeated at least three times. Bars and error bars refer to the mean ± SD. **** *p* < 0.0001 vs. control. (**B**) Representative images of the assay are shown in the figure.

To further examine the effect of **19** on the growth of cancer cells, a colony formation assay was conducted. Cultured cancer cells are capable of anchorage-independent growth, possessing the ability to grow in the absence of extracellular matrix and adjacent cells. This property of cancer cells has been linked to tumour cell aggressiveness in vivo [13]. Anchorage-independent growth may be assessed via a colony formation assay. Colony formation/clonogenic assays also detect the ability of cancer cells to survive a brief challenge with test agent and form progeny colonies; therefore, clonogenic assays were performed to investigate the effect of **19** on the ability of MDA-MB-468 and PANC-1 cells to form colonies (Figure 3). Results show that **19** was able to completely prevent the formation of colonies by the cancer cells after 24 h of treatment. These data indicate that **19** has the ability to kill MDA-MB-468 TNBC and PANC-1 pancreatic tumour-initiating cells and prevent tumour recolonisation, highlighting its cytotoxic potential.

**Figure 4:**
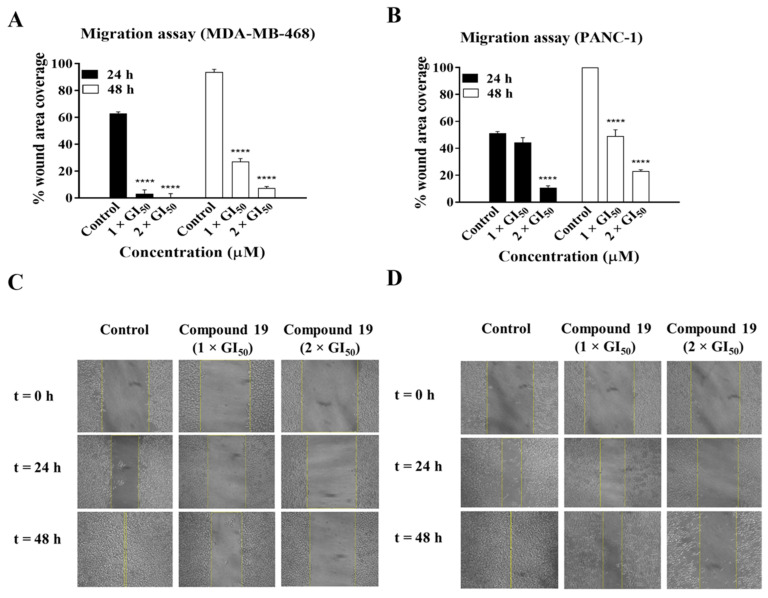
Compound **19** significantly inhibited the migration of MDA-MB-468 and PANC-1 cells after 24 h and 48 h. (**A**,**B**) The migration assay was conducted by forming a “wound” across a layer of cells followed by treatment with either solvent control or **19** at concentrations of 1 × GI_50_ and 2 × GI_50_. Cell migration was calculated and expressed as the percentage of “wound” area covered by the cells to the initial cell-free “wound” area. The assay was repeated at least three times. Bars and error bars refer to the mean ± SD. **** *p* < 0.0001 vs. control. (**C**,**D**) Representative microscopic images from one of the migration assay experiments.

We subsequently investigated the effect of **19** on cancer cell migration. Cancer cells exhibit the ability to metastasise or migrate from their site of origin to other organs within the body, which leads to the spread of cancer. Therefore, wound healing migration assays were performed to examine the effect of **19** on the migration of MDA-MB-468 and PANC-1 cells (Figure 4). Migration of MDA-MB-468 cells was reduced by ~3-fold and 13-fold, while that of PANC-1 cells was reduced by ~2-fold and ~4-fold relative to the control after 48 h of treatment with **19** at concentrations of 1 × GI_50_ and 2 × GI_50_, respectively. Therefore, these results indicate that **19** exhibits significant inhibitory effects on the migration of MDA-MB-468 and PANC-1 cells with a more pronounced effect on the former. The migration ability of cancer cells is a crucial factor in invasion and metastasis [14]; therefore, these results and those of the clonogenic assay reflect the potential of **19** to reduce or inhibit cancer cell survival, invasion, and metastasis in patients.

### 2.2. Compound ***19*** Induced Gap 2 (G2)/Mitosis (M) Phase Cell-Cycle Arrest by Triggering DNA Damage in MDA-MB-468 and PANC-1 Cancer Cells

**Figure 5:**
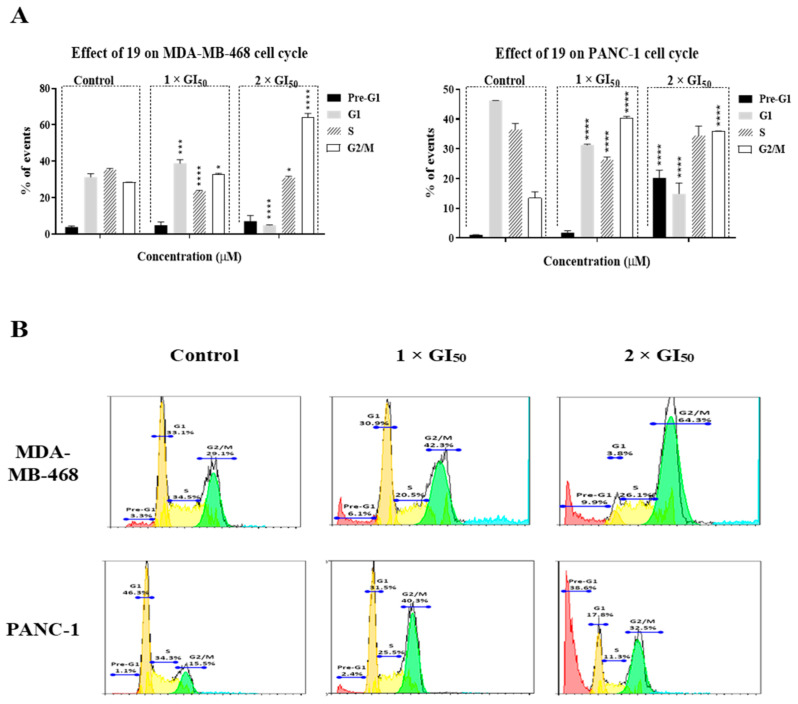
(**A**) Compound **19** induced gap 2 (G2)/mitosis (M) phase cell-cycle arrest in MDA-MB-468 and PANC-1 cells after 24 h of treatment at concentrations of 1 × GI_50_ and 2 × GI_50_. The experiment was repeated at least three times. Bars and error bars refer to mean ± SD. * *p* < 0.05 vs. control, *** *p* < 0.001 vs. control, **** *p* < 0.0001 vs. control. (**B**) Representative cell-cycle histograms from one of the experiments showing the effect of **19** on cell-cycle progression in MDA-MB-468 and PANC-1 cells after 24 h of treatment at concentrations of 1 × GI_50_ and 2 × GI_50_.

To further characterise the cytotoxic and growth inhibitory effect of **19** against cancer cells, cell-cycle analysis was conducted in order to investigate the effect of our compound on cell-cycle progression. MDA-MB-468 and PANC-1 treated with **19** at concentrations of 1 × GI_50_ and 2 × GI_50_ for 24 h showed a significant increase in the fraction of cells in the G2/M phase (Figure 5). Compound **19** induced the highest accumulation of cells in the G2/M phase at a concentration of 2 × GI_50_ for MDA-MB-468 cells (64.3% compared to 29.1% in the untreated cells). However, in PANC-1 cells, the highest accumulation of cells at the G2/M phase was induced at a concentration of 1 × GI_50_ (40.3% compared to 15.5% in the untreated cells), while increasing the treatment concentration to 2 × GI_50_ resulted in significantly increased numbers of events in G2/M and pre-G1 phases. These observations indicate that **19**, at 2 × GI_50_, causes apoptosis in a more pronounced manner in PANC-1 cells than in MDA-MB-468 cells.

**Figure 6:**
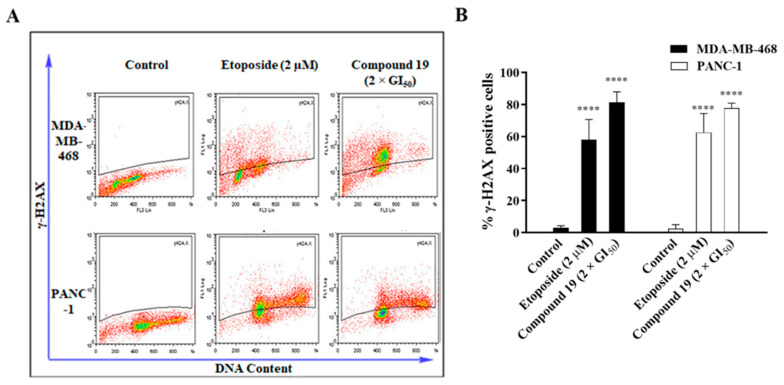
(**A**) Representative dot plots illustrating the formation of γ-H2AX in MDA-MB-468 and PANC-1 cells after 24 h of treatment with etoposide and **19** at concentrations of 2 µM and 2 × GI_50_, respectively. (**B**) The experiment was repeated three times. Bars and error bars refer to the mean ± SD. **** *p* < 0.0001 vs. control.

The induction of G2/M phase cell-cycle arrest by certain agents often indicates that they also cause DNA damage [15]. Therefore, since compound **19** was able to induce G2/M phase cell-cycle arrest, we investigated its ability to cause DNA damage. The formation of DNA double-strand breaks (DSB) as a result of DNA damage, is accompanied by phosphorylation of histone H2AX to γ-H2AX [16]; thus, DNA damage may be assessed via quantification of γ-H2AX. The induction of DNA damage by compound **19** was assessed by quantifying γ-H2AX-expressing cells post treatment via flow cytometry. Results showed that **19** (2 × GI_50_) was able to significantly increase the formation of γ-H2AX by ~8-fold in both MDA-MB-468 and PANC-1 cells relative to the control (Figure 6). Moreover, it is interesting to note that **19** also resulted in the formation of γ-H2AX at higher levels than the positive control, etoposide (2 µM). This clearly indicates that the observed cytotoxic activity of **19** involves the induction of DNA damage in the cancer cells.

### 2.3. Compound ***19*** Causes Severe Disruption in Cytoskeletal Structure

**Figure 7:**
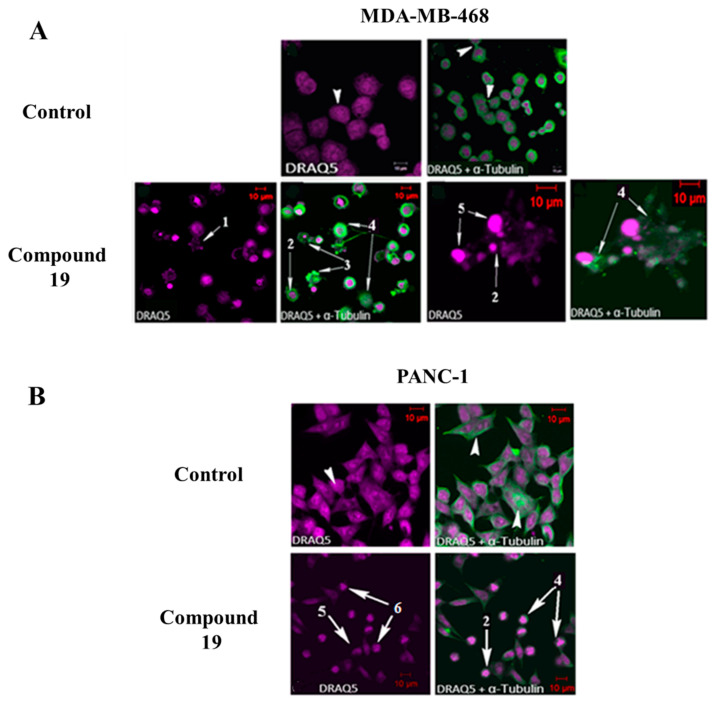
Effect of **19** on (**A**) MDA-MB-468 and (**B**) PANC-1 cells, after 24 h of exposure at GI_50_ concentration. Compound **19** causes nuclear fragmentation (1), multinucleation (2), membrane blebbing (3), microtubule network disruption (4), membrane disruption of nuclei (5), lunate morphology of chromatin, and chromatin condensation (6). The experiments were repeated three times. Cells were immunostained with an antibody specific for α-tubulin (green) and counterstained with DRAQ5 (purple).

Cell-cycle analysis revealed that **19** caused G2/M phase arrest in MDA-MB-468 and PANC-1 cancer cells, and this could be indicative of microtubule disruption [17]. Therefore, in order to determine whether **19** affects the microtubule network, MDA-MB-468 and PANC-1 cells were treated for 24 h with **19** at GI_50_ concentration, stained with DRAQ5 and monoclonal anti α-tubulin antibody, and morphologically investigated using confocal microscopy. Confocal microscopy images (Figure 7) revealed that **19** was able to induce characteristic features in cancer cells that are typical of a microtubule disrupting agent (MDA), such as multinucleation, nuclear fragmentation, and microtubule network disruption [17,18]. Moreover, apoptosis-related morphological changes were also observed following treatment with **19**, such as membrane blebbing, chromatin condensation, and nuclear fragmentation. Therefore, these data suggest that **19** interferes with the cancer cells’ microtubule network, leading to G2/M phase cell-cycle arrest, which later results in cell death and growth inhibition.

### 2.4. Compound ***19*** Induced Caspase-Dependent Apoptosis and Reactive Oxygen Species (ROS) Generation in MDA-MB-468 and PANC-1 Cancer Cells

**Figure 8:**
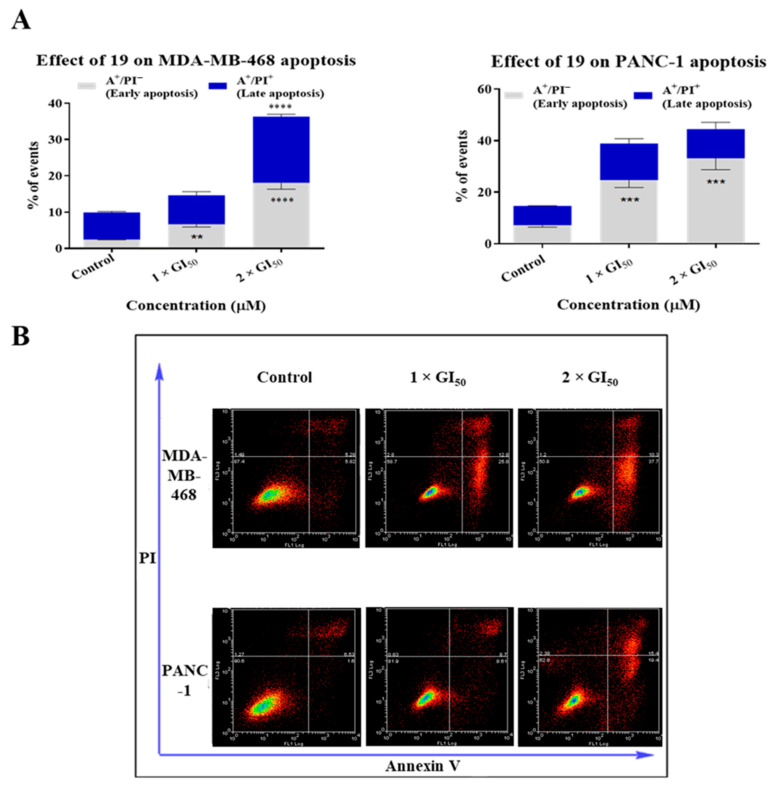
(**A**) Results of the annexin V/propidium iodide (PI) apoptosis assay showed that **19** induced apoptosis in MDA-MB-468 and PANC-1 cells after 24 h of treatment at concentrations of 1 × GI_50_ and 2 × GI_50_. Total apoptosis comprises both early and late apoptotic populations. The experiment was repeated at least three times. Bars and error bars refer to mean ± SD. ** *p* < 0.01 vs. control, *** *p* < 0.001 vs. control, **** *p* < 0.0001 vs. control. (**B**) Representative apoptosis quadrant plots illustrating the apoptotic effects of **19** on MDA-MB-468 and PANC-1 cells.

Cell-cycle analysis and confocal microscopy suggest that **19** causes apoptosis in MDA-MB-468 and PANC-1 cells. Therefore, to further confirm the induction of apoptosis by **19** in cancer cells, an annexin V/propidium iodide (PI) apoptosis assay was conducted. MDA-MB-468 and PANC-1 cells were treated with **19** for 24 h at concentrations of 1 × GI_50_ and 2 × GI_50_, and apoptotic populations were confirmed by annexin V-FITC/PI dual staining. Results show that **19** caused significant dose-dependent early apoptosis (A+/PI−) along with an insignificant number of necrotic cells in MDA-MB-468 (6.61% early apoptotic cells at 1 × GI_50_ and 18.1% at 2 × GI_50_ compared to 2.41% in the untreated cells) and PANC-1 cells (24.7% early apoptotic cells at 1 × GI_50_ and 33.2% at 2 × GI_50_ compared to 7.2% in the untreated cells) (Figure 8). A small insignificant increase in late apoptotic events (A+/PI+) was observed in PANC-1 cells; however, MDA-MB-468 cells demonstrated a significant increase in late apoptotic cells after treatment with **19** at 2 × GI_50_ (36.3% late apoptotic cells compared to 9.92% in the untreated cells), and this may be a consequence of more rapid apoptosis onset at this concentration. The highest percentage of apoptotic cells at 2 × GI_50_ was ~36% and 44% for MDA-MB-468 and PANC-1 cells, respectively (consistent with cell-cycle observations).

**Figure 9:**
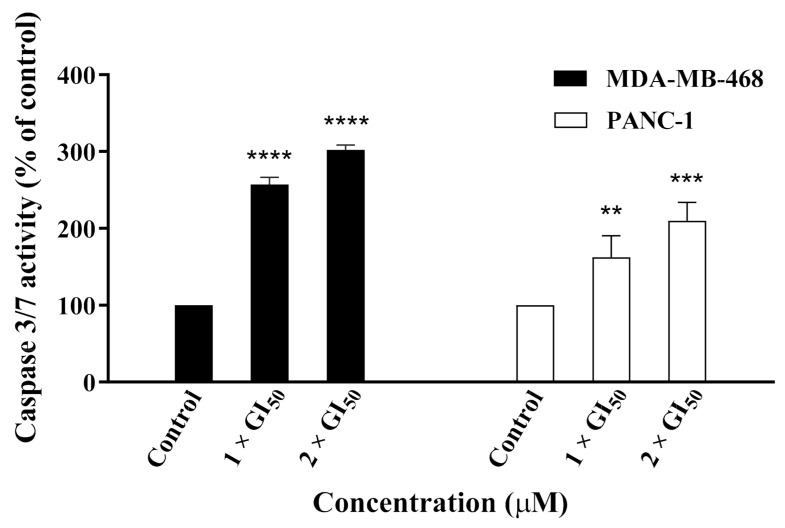
Caspase-3/7 activity of MDA-MB-468 and PANC-1 cells after 24 h of treatment with **19** at concentrations of 1 × GI_50_ and 2 × GI_50_. The experiment was repeated at least three times. Bars and error bars refer to the mean ± SD. ** *p* < 0.01 vs. control, *** *p* < 0.001 vs. control, **** *p* < 0.0001 vs. control.

We next explored whether caspases were involved in the induction of apoptosis by **19**, especially crucial executioner caspase 3, whose activation is considered to be a hallmark of apoptosis [19]. Therefore, MDA-MB-468 and PANC-1 cells were treated with **19** and caspase-Glo 3/7 assays were conducted to investigate the compound’s effect on caspase activation. Results showed that **19** was able to induce caspase-3/7 activation in a dose-dependent manner in both MDA-MB-468 and PANC-1 cells, with more pronounced activity observed in the former (Figure 9). This suggested that **19** exerted its cytotoxic activity, at least in part, via the induction of caspase-dependent apoptosis.

**Figure 10:**
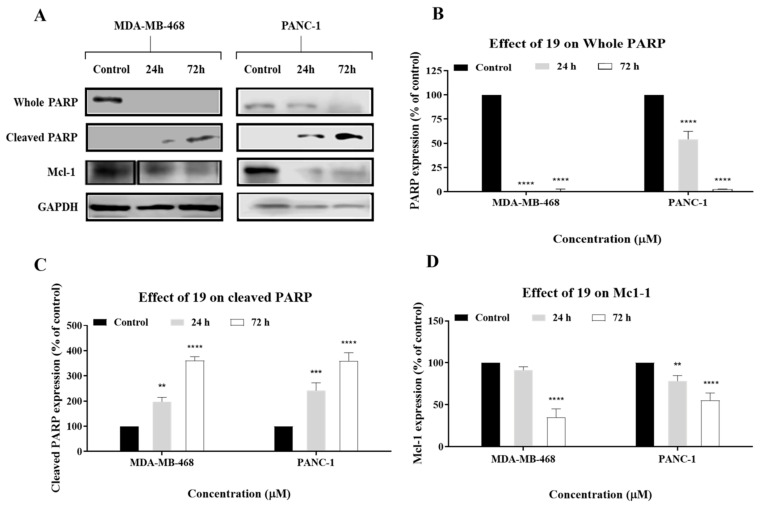
(**A**) Western blot analysis of PARP, cleaved PARP, and Mcl-1 in MDA-MB-468 and PANC-1 cells. Cells were treated with **19** for 24 h and 72 h at a concentration of 2 × GI_50_. GAPDH was used as an internal control. (**B**–**D**) Collated densitometric measurement of protein expression levels in MDA-MB-468 and PANC-1 cells. The experiment was repeated at least three times. Bars and error bars refer to the mean ± SD. ** *p* < 0.01 vs. control, *** *p* < 0.001 vs. control, **** *p* < 0.0001 vs. control.

Apoptosis can be further confirmed by investigating the expression levels of apoptosis-related proteins such as PARP and Mcl-1. PARP is a substrate of caspase-3 which plays an active role in a variety of key biological processes, and its cleavage is indicative of apoptosis [20]. Mcl-1 is an antiapoptotic Bcl-2 family protein. Therefore, MDA-MB-468 and PANC-1 cells were treated with **19**, and the expression levels of PARP, cleaved PARP, and Mcl-1 were analysed via Western blot. Results of Western blot analyses showed that **19** cleaved PARP and downregulated Mcl-1 in a time-dependent manner, which indicates that it induced apoptosis in the cancer cells (Figure 10). It is also crucial to note that PARP cleavage and Mcl-1 downregulation were more evident in PANC-1 cells than MDA-MB-468 cells, which might explain the higher percentage of apoptotic cells in the former that was observed in the cell-cycle analysis and annexin V/propidium iodide (PI) assays. Cleavage of PARP is also indicative of caspase activation, which corroborates the caspase assay results. In conclusion, the annexin V-FITC/PI assay, caspase assay, and Western blot analysis indicate that **19** induces apoptosis in MDA-MB-468 and PANC-1 cells.

**Figure 11:**
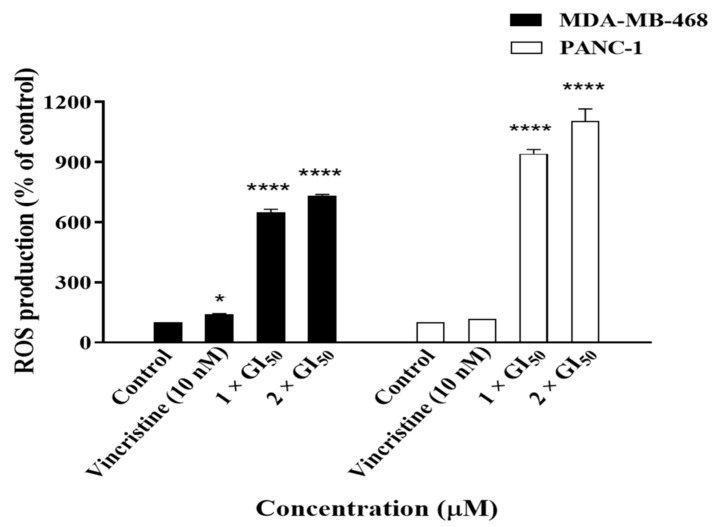
ROS production in MDA-MB-468 and PANC-1 cells after 24 h of treatment with Vincristine (10 nM) and **19** at concentrations of 1 × GI_50_ and 2 × GI_50_. The experiment was repeated at least three times. Bars and error bars refer to the mean ± SD. * *p* < 0.05 vs. control, **** *p* < 0.0001 vs. control.

After confirming the induction of apoptosis by **19**, we wanted to investigate the possibility that it was triggered via reactive oxygen species (ROS), as ROS are generated by many natural product-derived antitumour agents [21] and may damage DNA and play a role in apoptosis induction. Therefore, cells were treated with **19**, and ROS production was measured using a ROS-Glo H_2_O_2_ luminescence assay. Compound **19** significantly increased ROS production in MDA-MB-468 and PANC-1 cells by ~7- and 10-fold, relative to the untreated control, respectively (Figure 11). Increasing the treatment concentration from 1 × GI_50_ to 2 × GI_50_ did not result in further significant increases in ROS production in either cell line. It is also worth noting that ROS production as a result of treatment with **19** was much higher than ROS produced due to the positive control Vincristine in MDA-MB-468 and PANC-1 cells. Hence, it can be deduced that the induction of apoptosis by **19** in MDA-MB-468 and PANC-1 cells might be meditated, at least in part, via the production of ROS. However, further experiments are required to confirm the relationship between ROS production and apoptosis induction by **19** in the cancer cells.

### 2.5. Compound ***19*** Exerted Its Cytotoxic Activity in MDA-MB-468 and PANC-1 Cells via Inhibition of Akt/4EBP1 Signalling and Downregulation of c-Myc

Several studies have reported that cardamonin exerts its cytotoxic activity via inhibition of mTOR signalling [22,23,24]. A further study showed that cardamonin reduced the expression level of phosphorylated Akt, which is an upstream regulator of mTOR [25]. The Akt signalling pathway is normally involved, via the PI3K/Akt/mTOR pathway, in cell proliferation and survival. However, this pathway is abnormally activated in several types of breast cancer, including TNBC, which contributes to the disease’s aggressiveness [26]. Akt activation was also often observed in pancreatic cancer, whereby it is considered as a “master regulator” of cancer cell metastasis [27].

**Figure 12:**
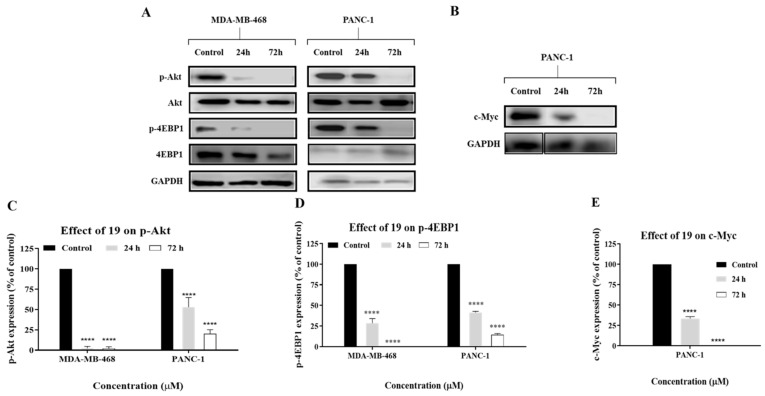
(**A**,**B**) Western blot analysis of p-Akt, Akt, p-4EBP1, 4EBP1, and c-Myc. MDA-MB-468 and PANC-1 cells were treated with **19** for 24 h and 72 h at a concentration of 2 × GI_50_. GAPDH was used as an internal control. (**C**–**E**) Collated densitometric measurement of protein expression levels in MDA-MB-468 and PANC-1 cells. The experiment was repeated at least three times. Bars and error bars refer to the mean ± SD. **** *p* < 0.0001 vs. control.

Therefore, we reasoned that the observed cytotoxic activity of **19**, an analogue of cardamonin, against MDA-MB-468 and PANC-1 cells might have occurred via a similar mechanism to that of cardamonin. Western blot analysis showed that **19** decreased the expression of phosphorylated Akt (p-Akt) in a time-dependent manner in both MDA-MB-468 and PANC-1 cells (Figure 12). Moreover, results have shown that **19** also downregulated the expression levels of phosphorylated 4EBP1 (p-4EBP1), and this protein is the first downstream substrate of mTOR. Therefore, these results suggest that **19** exerted its cytotoxic activity in MDA-MB-468 and PANC-1 cells, at least in part, via inhibition of the Akt signalling pathway. It can be further deduced that **19** is involved in the modulation of the mTOR signalling pathway, as our data clearly show that it downregulated p-Akt and p-4EBP1, which are upstream and downstream components of mTOR, respectively.

TNBC and pancreatic cancer are also characterised by an overexpression of the oncoprotein c-Myc, which contributes to the growth and proliferation of cancer cells [28,29]. Therefore, we wanted to investigate if the cytotoxic activity of **19** involved suppression of c-Myc expression. Western blot analysis showed that **19** downregulated the expression level of c-Myc in PANC-1 cells (Figure 12); however, no change was observed in MDA-MB-468 cells (result not shown). These results are consistent with c-Myc’s role in the promotion of pancreatic malignancy and pancreatic cancer cell proliferation and migration, and they concur with findings of previous studies which reported that inhibition of c-Myc affects the progression of pancreatic cancer cells [30,31,32]. This suggests that **19** may perturb multiple signalling nodes pertinent to tumourigenesis and can exert its cytotoxic activity in PANC-1 cells via a different/additional mechanism than that of MDA-MB-468.

## 3. Materials and Methods

### 3.1. Synthesis of ***19***

Compound **19** was synthesised, and its structure was fully characterised as reported in our previous study [11].

### 3.2. Cell Culture

Compound **19** was tested against MDA-MB-468 (TNBC) and PANC-1 (pancreatic) cancer cell lines. Cell cultures were maintained using RPMI 1640 (Sigma-Aldrich, Gillingham, UK) supplemented with sodium bicarbonate (2 g/L, Sigma-Aldrich, Gillingham, UK), 1% 200 mM l-glutamine (Sigma-Aldrich, Gillingham, UK), and heat-inactivated 10% foetal bovine serum (FBS) (Sigma-Aldrich, Gillingham, UK) under a humidified atmosphere containing 5% CO_2_ in air at 37 °C.

### 3.3. Cell Viability Assay

The spectrophotometric MTT assay was used to measure cell viability. Cells were seeded in 96-well plates at a density of 3 × 10^3^ cells per well and incubated for 24 h at 37 °C prior to treatment. Cells were then treated with **19** at different concentrations and incubated for 72 h. After incubation, MTT solution was added to each well, and the resulting absorbance was read.

### 3.4. Colony Formation Assay

The assay was performed as previously reported [33].

### 3.5. Migration Assay

Cells were seeded in six-well plates at a density of 1 × 10^6^ cells per well and allowed to grow until confluency. This was followed by forming a scratch or “wound” in the layer of cells using a sterile 200 µL pipette tip. The cells were washed with phosphate-buffered saline (PBS) and incubated with **19** at a concentration of 2 × GI_50_ in the presence of 10% FBS for 48 h. Images of the cells were then acquired via an inverted microscope (Nikon ECLIPSE TS100, Tokyo, Japan) equipped with a camera at 0 h, 24 h, and 48 h. The images were analysed via ImageJ software (NIH, Bethesda, Maryland, USA).

### 3.6. Cell-Cycle Analysis

Cell-cycle analysis was performed according to a method that involves using a fluorochrome solution containing 50 µg/mL propidium iodide (PI), 0.1 mg/mL ribonuclease A, 0.1% *v/v* Triton X-100, and 0.1% *w/v* sodium citrate in *d*-H_2_O [11]. Cancer cells were seeded in six-well plates at a density of 0.5–1 × 10^6^ cells per well and treated for 24 h with **19** at concentrations of 1 × GI_50_ and 2 × GI_50_. Cells were then harvested, centrifuged, and resuspended in 0.3–0.5 mL of the fluorochrome solution and stored overnight in the dark at 4 °C. Lastly, a flow cytometer (Beckman Coulter FC500 MCL, Indianapolis, IN, USA) was used to conduct the cell-cycle analysis, while the resulting data were analysed via Weasel flow cytometry analysis software (Version 3.5, WEHI, Melbourne, Australia).

### 3.7. γ-H2AX Detection for DNA Damage Assessment

Cells were seeded and allowed to adhere for 24 h, before treatment for 24 h with **19** and etoposide (positive control) at concentrations of 2 × GI_50_ and 2 µM, respectively. Cells were then harvested, fixed with 1% methanol free formaldehyde in PBS, centrifuged, resuspended in 200 μL of γ-H2AX 1° antibody (1:3333 dilution, EMD-Millipore, Darmstadt, Germany), and incubated at room temperature for 1.5 h. This was followed by addition of a secondary antibody (1:1750 dilution, goat anti-mouse Alexa Fluor 488, Invitrogen, CA, USA) and incubated at room temperature for 1 h. Cells were then washed and resuspended in 300 μL of 50 μg/mL propidium iodide/0.1 mg/mL RNAse A in PBS and later incubated. Measurements were finally taken via a flow cytometer (Beckman Coulter FC500 MCL, Indianapolis, IN, USA) and later analysed via Weasel flow cytometry analysis software (v. 3.5) (WEHI, Melbourne, VIC, Australia).

### 3.8. Confocal Microscopy

Cells were seeded in eight-well μ-slides at a density of 1 × 10^4^ cells per well in 200 μL of medium and incubated overnight. Cells were then treated with **19** for 24 h and later fixed with formaldehyde (3.7% in PBS; 10–15 min). Cells were permeabilised using PBT (PBS + 0.1% TritonX-100; 2–3 min). Cells were incubated/treated for 1 h with PBT and 1% bovine serum albumin (BSA) in order to prevent nonspecific protein binding. They were later incubated for 2 h with monoclonal anti-α-tubulin antibody (Thermo Scientific), washed with PBT, and incubated for 1 h with fluorescent secondary antibody (1:500 dilution, anti-mouse immunoglobulin G (IgG) Alexa Fluor 488 F, Invitrogen, CA, USA) in the dark. DNA-binding dye DRAQ5 (1:3000) was added and cells were incubated for 5 min in the dark. Lastly, visualisation of the cells and image capture was performed using a Zeiss LSM510 Meta confocal microscope conjugated with Zeiss LSM software [34].

### 3.9. Annexin V-FITC/Propidium Iodide (PI) Apoptosis Assay

Cells were seeded in 12-well plates at a density of 1.5 × 10^5^ cells per well and incubated overnight. Cells were then treated with **19** at concentrations of 1 × GI_50_ and 2 × GI_50_ for 24 h. Following treatment, cells were trypsinised, collected in FACS tubes, kept on ice for 10 min, washed with PBS, and centrifuged to form a pellet. Annexin V-FITC (5 μL) and 100 μL of 1 × annexin-V buffer were added to the cells, followed by incubation for 15 min at room temperature. PI (10 μL; 50 μg/mL in PBS) and 400 μL of annexin-V buffer were later added, and the cells were then placed on ice for 10 min prior to analysis using a flow cytometer (Beckman Coulter FC500 MCL, Indianapolis, IN, USA). Data were analysed via Weasel flow cytometry analysis software (WEHI, Melbourne, VIC, Australia).

### 3.10. Caspase-Glo 3/7 Assay

The assay was performed using the caspase-Glo 3/7 assay kit (Promega, Southampton, UK). Cancer cells were seeded at a density of 5 × 10^3^ cells per well and incubated overnight. The cells were then treated with **19** at concentrations of 1 × GI_50_ and 2 × GI_50_ for 24 h. This was followed by addition of the caspase-Glo 3/7 reagent and incubation for 60 min prior to reading the results via an Envision 2104 multi-label plate reader (PerkinElmer, Waltham, MA, USA).

### 3.11. Western Blotting Analysis

Cells were seeded in dishes at a density of 1–2 × 10^6^ per dish. After 24 h, cells were treated with **19** at a concentration of 2 × GI_50_ for 24 h and 72 h. Following treatment, cell lysates were prepared, and protein concentrations were calculated using the Bradford assay. Protein was subjected to SDS-PAGE for separation and transferred to the nitrocellulose membrane. Whole PARP, cleaved PARP, Mcl-1, Akt, p-Akt, 4EBP1, p-4EBP1, c-Myc, and GAPDH primary antibodies were purchased from Cell Signalling Technologies (Danvers, MA, USA). Anti-rabbit and anti-mouse immunoglobulin G (IgG) horseradish peroxidase-conjugated secondary antibodies were obtained from Dako (Santa Clara, CA, USA). Densitometric analysis was conducted using ImageJ software (NIH, MD, USA).

### 3.12. Reactive Oxygen Species (ROS) Detection

The ROS-Glo H_2_O_2_ luminometric-based assay (Promega, Southampton, UK) was used to detect the production of ROS. Cancer cells were seeded at a density of 5 × 10^3^ cells per well and incubated overnight. The cells were then treated with **19** at concentrations of 1 × GI_50_ and 2 × GI_50_ for 24 h. H_2_O_2_ substrate (25 μM) was then added to the cells followed by the addition of 100 μL of ROS-Glo detection solution, and samples were then incubated for 20 min at room temperature. Resulting luminescence was detected using an Envision 2104 multilabel plate reader (PerkinElmer, Waltham, MA, USA).

### 3.13. Statistical Analysis

Experiments were generally repeated ≥3 times and data were expressed as the mean ± SD. Experimental data were analysed via Graphpad Prism software (Version 7.04, Graphpad Software Inc, San Diego, CA, USA). Statistical differences among means were analysed by one-way and two-way ANOVA followed by Dunnett’s multiple-comparison tests. Data represent significant values as follows: * *p* < 0.05, ** *p* < 0.01, *** *p* < 0.001, and **** *p* < 0.0001.

## 4. Conclusions

**Figure 13:**
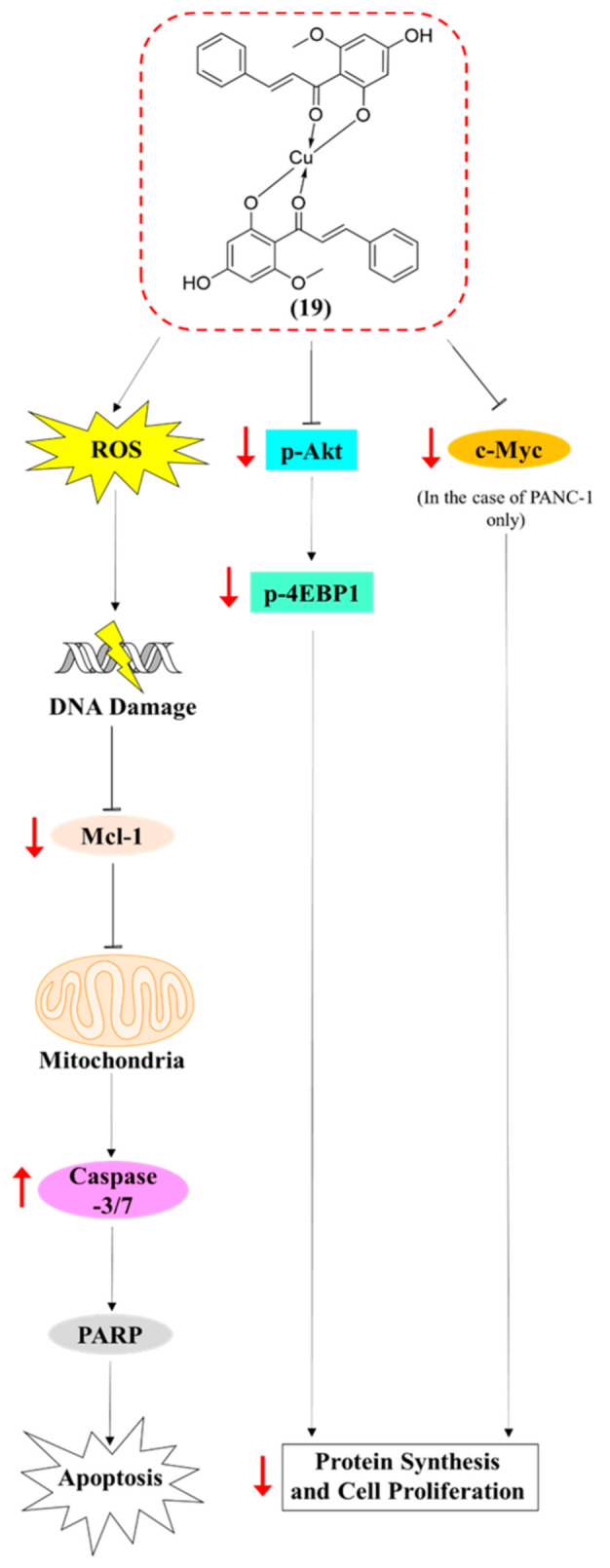
Schematic representation of the proposed signalling pathways perturbed by **19** in MDA-MB-468 and PANC-1 cells.

In summary, the present study involved a detailed assessment of the cytotoxic activity of novel semi-synthetic compound **19**, investigating its activity and molecular mechanisms of action in MDA-MB-468 and PANC-1 cell lines. Compound **19** demonstrated significant cytotoxic activity against the cancer cell lines and showed higher bioactivity than cardamonin, which reflects the positive effect of complexation on bioactivity in this case. Furthermore, **19** inhibited cancer cell colony formation and migration ability. After establishing the efficacy of **19,** we next explored its mode(s) of action, whereby it was found that our compound increased ROS production, which may have resulted in the observed induction of DNA damage and subsequent G2/M phase cell-cycle arrest. It was also found that **19** induced caspase-dependent apoptosis in MDA-MB-468 and PANC-1 cells, with a more pronounced effect on the latter, and this induced apoptosis could be due to the production of ROS. Further mechanistic studies showed that **19** inhibited Akt signalling. Figure 13 summarises the proposed signalling pathways triggered by compound **19** in the cancer cells. Taken together, this study further highlighted the anticancer potential of **19**, corroborating the findings of our previous study [11], and it provides a better understanding of the mode(s) of action of our compound. To the best of our knowledge, compound **19** is the first synthetic cardamonin analogue that has demonstrated promising antitumour activity and the only analogue whose mechanism of action has been studied in such detail, as presented in this study. It is expected that such groundwork will guide future in vivo studies on **19** in order to further develop the compound as an effective anticancer drug for TNBC and pancreatic cancer.

## Figures and Tables

**Table 1:** GI_50_ values of **19** against MDA-MB-468, PANC-1, and MRC-5 cells.

Compound	GI_50_ (µM) ^1^			Selectivity Index (SI) ^2^	
	MDA-MB-468	PANC-1	MRC-5	MDA-MB-468	PANC-1
**19**	6.14 ± 0.06	12.48 ± 0.70	32.60 ± 5.13	5.3	2.6
**Cardamonin**	34.33 ± 0.55	19.21 ± 2.37	43.25 ± 3.31	1.2	2.2
**Vincristine Sulphate**	0.03 ± 0.0005	0.48 ± 0.24	5.58 ± 0.38	186	11.6

^1^ GI_50_ values are reported as the mean (GI_50_ ± SD) of at least three independent experiments. ^2^ SI = (GI_50_ of MRC-5)/(GI_50_ of MDA-MB-468 or PANC-1).

## Data Availability

The data presented in this study are available upon request from the corresponding author.

## Sample Availability

Samples of the compound are available from the authors.

## References

[1] Cancer. https://www.who.int/news-room/fact-sheets/detail/cancer.

[2] Worldwide Cancer Incidence Statistics. https://www.cancerresearchuk.org/health-professional/cancer-statistics/worldwide-cancer/incidence.

[3] Bianchini G., Balko J.M., Mayer I.A., Sanders M.E., Gianni L. (2016). Triple-negative breast cancer: Challenges and opportunities of a heterogeneous disease. Nat. Rev. Clin. Oncol..

[4] Kleeff J., Korc M., Apte M., La Vecchia C., Johnson C.D., Biankin A.V., Neale R.E., Tempero M., Tuveson D.A., Hruban R.H. (2016). Pancreatic Cancer. Nat. Rev. Dis. Prim..

[5] Grant C.V., Carver C.M., Hastings S.D., Ramachandran K., Muniswamy M., Risinger A.L., Beutler J.A., Mooberry S.L. (2019). Triple-negative breast cancer cell line sensitivity to englerin A identifies a new, targetable subtype. Breast Cancer Res. Treat..

[6] Gonçalves L.M., Valente I.M., Rodrigues J.A. (2014). An overview on cardamonin. J. Med. Food.

[7] Break M.K.B., Chiang M., Wiart C., Chin C.-F., Khoo A.S.B., Khoo T.-J. (2021). Cytotoxic activity of boesenbergia rotunda extracts against nasopharyngeal carcinoma cells (HK1). cardamonin, a boesenbergia rotunda constituent, inhibits growth and migration of HK1 cells by inducing caspase-dependent apoptosis and G2/M-phase arrest. Nutr. Cancer.

[8] Shrivastava S., Jeengar M.K., Thummuri D., Koval A., Katanaev V.L., Marepally S., Naidu V.G.M. (2017). Cardamonin, a chalcone, inhibits human triple negative breast cancer cell invasiveness by downregulation of Wnt/β-catenin signaling cascades and reversal of epithelial-mesenchymal transition. BioFactors.

[9] Nawaz J., Rasul A., Shah M.A., Hussain G., Riaz A., Sarfraz I., Zafar S., Adnan M., Khan A.H., Selamoglu Z. (2020). Cardamonin: A new player to fight cancer via multiple cancer signaling pathways. Life Sci..

[10] Kong W., Li C., Qi Q., Shen J., Chang K. (2020). Cardamonin induces G2/M arrest and apoptosis via activation of the JNK-FOXO3a pathway in breast cancer cells. Cell Biol. Int..

[11] Break M.K.B., Hossan M.S., Khoo Y., Qazzaz M.E., Al-Hayali M.Z.K., Chow S.C., Wiart C., Bradshaw T.D., Collins H., Khoo T.-J. (2018). Discovery of a highly active anticancer analogue of cardamonin that acts as an inducer of caspase-dependent apoptosis and modulator of the MTOR pathway. Fitoterapia.

[12] Shahraki S., Saeidifar M., Delarami H.S., Kazemzadeh H. (2020). Molecular docking and inhibitory effects of a novel cytotoxic agent with bovine liver catalase. J. Mol. Struct..

[13] Mori S., Chang J.T., Andrechek E.R., Matsumura N., Baba T., Yao G., Kim J.W., Gatza M., Murphy S., Nevins J.R. (2009). Anchorage-independent cell growth signature identifies tumors with metastatic potential. Oncogene.

[14] Nandakumar N., Muthuraman S., Gopinath P., Nithya P., Gopas J., Kumar R.S. (2017). Synthesis of coumaperine derivatives: Their NF-ΚB inhibitory effect, inhibition of cell migration and their cytotoxic activity. Eur. J. Med. Chem..

[15] Wu C.-F., Efferth T. (2015). Miltirone Induces G2/M cell cycle arrest and apoptosis in CCRF-CEM acute lymphoblastic leukemia cells. J. Nat. Prod..

[16] Sone K., Piao L., Nakakido M., Ueda K., Jenuwein T., Nakamura Y., Hamamoto R. (2014). Critical role of lysine 134 methylation on histone H2AX for γ-H2AX production and DNA repair. Nat. Commun..

[17] Hossan M.S., Chan Z.-Y., Collins H.M., Shipton F.N., Butler M.S., Rahmatullah M., Lee J.B., Gershkovich P., Kagan L., Khoo T.-J. (2019). Cardiac glycoside cerberin exerts anticancer activity through PI3K/AKT/MTOR signal transduction inhibition. Cancer Lett..

[18] Smedley C.J., Stanley P.A., Qazzaz M.E., Prota A.E., Olieric N., Collins H., Eastman H., Barrow A.S., Lim K.-H., Kam T.-S. (2018). Sustainable syntheses of (−)-jerantinines A & E and structural characterisation of the jerantinine-tubulin complex at the colchicine binding site. Sci. Rep..

[19] Gong L., Tang Y., An R., Lin M., Chen L., Du J. (2017). RTN1-C mediates cerebral ischemia/reperfusion injury via ER stress and mitochondria-associated apoptosis pathways. Cell Death Dis..

[20] Vizetto-Duarte C., Custódio L., Gangadhar K.N., Lago J.H.G., Dias C., Matos A.M., Neng N., Nogueira J.M.F., Barreira L., Albericio F. (2016). Isololiolide, a carotenoid metabolite isolated from the brown alga cystoseira tamariscifolia, is cytotoxic and able to induce apoptosis in hepatocarcinoma cells through caspase-3 activation, decreased Bcl-2 levels, increased P53 expression and PARP cleavage. Phytomedicine.

[21] Tsang W.P., Chau S.P.Y., Kong S.K., Fung K.P., Kwok T.T. (2003). Reactive oxygen species mediate doxorubicin induced P53-independent apoptosis. Life Sci..

[22] Tang Y., Fang Q., Shi D., Niu P., Chen Y., Deng J. (2014). MTOR inhibition of cardamonin on antiproliferation of A549 cells is involved in a FKBP12 independent fashion. Life Sci..

[23] Niu P., Li J., Chen H., Zhu Y., Zhou J., Shi D. (2020). Anti-proliferative effect of cardamonin on MTOR inhibitor-resistant cancer cells. Mol. Med. Rep..

[24] Xue Z.-G., Niu P.-G., Shi D.-H., Liu Y., Deng J., Chen Y.-Y. (2015). Cardamonin inhibits angiogenesis by MTOR downregulation in SKOV3 cells. Planta Med..

[25] Zhou X., Zhou R., Li Q., Jie X., Hong J., Zong Y., Dong X., Zhang S., Li Z., Wu G. (2019). Cardamonin inhibits the proliferation and metastasis of non-small-cell lung cancer cells by suppressing the PI3K/Akt/MTOR pathway. Anticancer Drugs.

[26] Costa R.L.B., Han H.S., Gradishar W.J. (2018). Targeting the PI3K/AKT/MTOR pathway in triple-negative breast cancer: A review. Breast Cancer Res. Treat..

[27] Wei R., Cortez Penso N.E., Hackman R.M., Wang Y., Mackenzie G.G. (2019). Epigallocatechin-3-gallate (EGCG) suppresses pancreatic cancer cell growth, invasion, and migration partly through the inhibition of Akt pathway and epithelial-mesenchymal transition: Enhanced efficacy when combined with gemcitabine. Nutrients.

[28] Hu M.-H., Wu T.-Y., Huang Q., Jin G. (2019). New substituted quinoxalines inhibit triple-negative breast cancer by specifically downregulating the c-MYC transcription. Nucleic Acids Res..

[29] Skoudy A., Hernández-Muñoz I., Navarro P. (2011). Pancreatic ductal adenocarcinoma and transcription factors: Role of c-Myc. J. Gastrointest. Cancer.

[30] Buchholz M., Schatz A., Wagner M., Michl P., Linhart T., Adler G., Gress T.M., Ellenrieder V. (2006). Overexpression of C-Myc in pancreatic cancer caused by ectopic activation of NFATc1 and the Ca^2+^/calcineurin signaling pathway. EMBO J..

[31] Yu Q., Zhou X., Xia Q., Shen J., Yan J., Zhu J., Li X., Shu M. (2016). Long non-coding RNA CCAT1 that can be activated by c-Myc promotes pancreatic cancer cell proliferation and migration. Am. J. Transl. Res..

[32] Sodir N.M., Kortlever R.M., Barthet V.J.A., Campos T., Pellegrinet L., Kupczak S., Anastasiou P., Brown Swigart L., Soucek L., Arends M.J. (2020). Myc instructs and maintains pancreatic adenocarcinoma phenotype. Cancer Discov..

[33] Qazzaz M.E., Raja V.J., Lim K.-H., Kam T.-S., Lee J.B., Gershkovich P., Bradshaw T.D. (2016). In vitro anticancer properties and biological evaluation of novel natural alkaloid jerantinine B. Cancer Lett..

[34] Collins H.M., Abdelghany M.K., Messmer M., Yue B., Deeves S.E., Kindle K.B., Mantelingu K., Aslam A., Winkler G.S., Kundu T.K. (2013). Differential effects of garcinol and curcumin on histone and P53 modifications in tumour cells. BMC Cancer.

